# An inclusive multivariate approach to neural localization of language components

**DOI:** 10.1007/s00429-024-02800-9

**Published:** 2024-05-02

**Authors:** William W. Graves, Hillary J. Levinson, Ryan Staples, Olga Boukrina, David Rothlein, Jeremy Purcell

**Affiliations:** 1 Department of Psychology, Rutgers University, Smith Hall, Room 301, 101 Warren Street, Newark, NJ 07102 USA; 2 Georgetown University Medical Center, Washington, DC USA; 3 Kessler Foundation, West Orange, NJ USA; 4 VA Boston Healthcare System, Boston, MA USA; 5 University of Maryland, College Park, MD USA

**Keywords:** Functional magnetic resonance imaging, Reading, Language, Multivariate pattern analysis, Representational similarity analysis

## Abstract

To determine how language is implemented in the brain, it is important to know which brain areas are primarily engaged in language processing and which are not. Existing protocols for localizing language are typically univariate, treating each small unit of brain volume as independent. One prominent example that focuses on the overall language network in functional magnetic resonance imaging (fMRI) uses a contrast between neural responses to sentences and sets of pseudowords (pronounceable nonwords). This contrast reliably activates peri-sylvian language areas but is less sensitive to extra-sylvian areas that are also known to support aspects of language such as word meanings (semantics). In this study, we assess areas where a multivariate, pattern-based approach shows high reproducibility across multiple measurements and participants, identifying these areas as multivariate regions of interest (mROI). We then perform a representational similarity analysis (RSA) of an fMRI dataset where participants made familiarity judgments on written words. We also compare those results to univariate regions of interest (uROI) taken from previous sentences > pseudowords contrasts. RSA with word stimuli defined in terms of their semantic distance showed greater correspondence with neural patterns in mROI than uROI. This was confirmed in two independent datasets, one involving single-word recognition, and the other focused on the meaning of noun-noun phrases by contrasting meaningful phrases > pseudowords. In all cases, areas of spatial overlap between mROI and uROI showed the greatest neural association. This suggests that ROIs defined in terms of multivariate reproducibility can help localize components of language such as semantics. The multivariate approach can also be extended to focus on other aspects of language such as phonology, and can be used along with the univariate approach for inclusively mapping language cortex.

**Supplementary Information:**

The online version contains supplementary material available at 10.1007/s00429-024-02800-9.

## Introduction

The use of language is a fundamental human ability, and its impairment has major consequences for quality of life. Knowing what areas of the brain are carrying out language-related functions, and what those functions are, is critically important for understanding how language works in the brain. From a basic science perspective, it is important to know if a patch of cortex being investigated represents some aspect of language, what that aspect is, and whether it is spatially distinct from, or co-localizes with, other aspects of cognition. For example, fundamental aspects of everyday language include reading words and retrieving names for objects. Impairment of these abilities can lead to difficulty acquiring written information or conveying content in conversation. In cases where surgical excision of brain tissue is required to treat a clinical condition, it is critically important during pre-surgical planning to know what brain areas house language representations. As a prerequisite for making an improved and clinically useful language localizer that highlights multiple critical aspects of language in the brain, we must first gain a more complete understanding of where those areas are and how reliably they can be detected.

To gain a more detailed knowledge of language areas in the brain, we must address the fact that language is not monolithic. Rather, it is at minimum a combination of phonological (auditory form), syntactic (grammatical forms), semantic (meaning), and in the case of written language, orthographic (visual form) processing. The exact neural distribution of these functions is not yet known. While it is clear that different functions are spatially distributed in different parts of the brain, especially in primary sensory and motor cortices, the degree and distribution of such modularity in higher-order association cortex remains unclear (Binder and Desai 2011; Lambon Ralph et al. 2017; Meyer and Damasio 2009; Rockland and Graves 2023; Smallwood et al. 2021). A view of language as exclusively areas responding to sentences more than pseudowords (Fedorenko et al. 2010), for example, would lead to an underestimation of areas for phonology, as both words and pseudowords contain valid phonological forms. Indeed, naming and word retrieval deficits related to difficulties retrieving word forms are widespread following left temporal lobectomy (Langfitt and Rausch 1996; Pauli et al. 2017), presumably reflecting poor pre-surgical localization of relevant critical neural tissue.

Beyond acknowledging the multi-componential nature of language, localizing those components in the brain also faces challenges of reliability and reproducibility. While functional magnetic resonance imaging (fMRI) was initially hailed as a promising candidate for replacing the hemisphere-level localization available from the intracarotid sodium amobarbital (Wada) test (Swanson et al. 2007), advances in neuroimaging analysis methods have raised questions even about its within-subject reproducibility (Agarwal et al. 2019; Benke et al. 2006; Wilson et al. 2017). Indeed, issues with reproducibility likely contribute to the gap between the promise of fMRI to enhance the translation of basic science findings into treatments and its spotty record of actually doing so (O’Connor and Zeffiro 2019).

To achieve the longer-term aim of a clinically useful mapping of crucial language components in the brain, we must first establish the combination of tasks, scanning protocols, and analyses required to reliably and reproducibly localize its critical components. Some progress has been made in this area in terms of providing specific pre-surgical language mapping protocols (Binder et al. 2008; Bookheimer 2007; Diachek et al. 2022; Thomas et al. 2023). However, acquired language deficits such as anomia continue to be widespread after neurosurgery for epilepsy and tumor removal (Binder et al. 2020; Hamberger 2015; Papagno et al. 2011, 2016). Here we lay the groundwork for using more current multivariate methods to detect areas that are reliably and reproducibly involved in language, including those beyond the basic peri-sylvian network.

### Current study

Compared to whole-brain analyses, using a localizer has the advantages of reducing the need to correct for multiple comparisons (thereby increasing sensitivity to detect an effect), and increasing specificity of the cognitive interpretation of areas activated in the localizer (Poldrack 2007; Saxe et al. 2006). A disadvantage is that it risks blinding the experimenter to potentially important effects occurring outside the localizer (Friston et al. 2006). To mitigate this disadvantage, the approach used here focuses on areas showing high *representational fidelity* (similarity structure that is reproducible across repetitions) for a condition of interest such as words or sentences, compared to a condition such as unpronounceable consonant strings or a simple fixation baseline that does not involve phonology. Regions localized in this way will be referred to as multivariate regions of interest (mROI). Such regions can then be queried for the presence and distribution of representations related to multiple aspects of language, such as semantics, syntax, or phonology. While the focus here is on language, and word-level semantics in particular, the representational fidelity measure was first worked out in the domain of attention (Rothlein et al. 2018). Indeed, our overall approach could be applied to any high-level cognitive domain thought to be composed of multiple sub-components, such as working memory or cognitive control.

After defining the mROI, we perform a whole-cortex representational similarity analysis (RSA; Kriegeskorte et al. 2008) to identify brain regions associated with word-level semantics. We then compare average parameter estimates within the mROI and the univariate region of interest (uROI). In our primary study (Study 1), we test the mROI using a semantic RSA analysis because the uROI was also largely defined in terms of semantics (the sentences > pseudoword contrast from Fedorenko et al. 2010). We hypothesize that the neural representations will be better revealed using multivariate pattern analysis. We use a partial correlation approach to RSA that statistically controls for stimulus-stimulus associations from two other sources: phonology and orthography. Compared to the uROI, we expect a more inclusive multivariate map that can be used to broadly define regions important for generally defined language function, but which also can be used to examine more nuanced, decomposable linguistic component parts.

To test our hypothesis that neural representations for semantics will be better revealed using multivariate pattern analysis, we performed a primary study (Study 1) to define the more inclusive language network, and then we considered data from two secondary studies to probe the generalizability and utility of this language mapping approach. Study 2 was a re-examination of data from a lexical decision task (Graves et al. 2017). Judging whether or not individual items are words is analogous to judging whether a word is familiar, so it was deemed a suitable independent dataset for testing the generalizability of the results. Study 3 involved meaningfulness judgments of noun-noun phrases (Graves, Binder, Graves et al. 2010a). This was chosen to test the additional hypothesis that univariate localizers, particularly those based on multi-word sequences, might be more suited to revealing activation in univariate analyses of multi-word stimuli.

Beyond activation or strength of neural association, lateralization has also been proposed as a test for face validity when using language tasks (Wilson et al. 2017). This is based in part on the consistent neuropsychological finding that left- but typically not right-hemisphere damage leads to difficulty with language (aphasia; Alexander 2003; Damasio 1992; Damasio 1998), as well as a meta-analysis of functional neuroimaging studies showing greater activation for semantics in left than right hemisphere (Binder et al. 2009). Therefore, we also test for lateralization of activation within mROI and uROI for all three studies. We hypothesize that, despite being more spatially inclusive than the uROI, results within the mROI will also show at least as much, if not more, left-lateralization.

## Methods

**Fig. 1:**
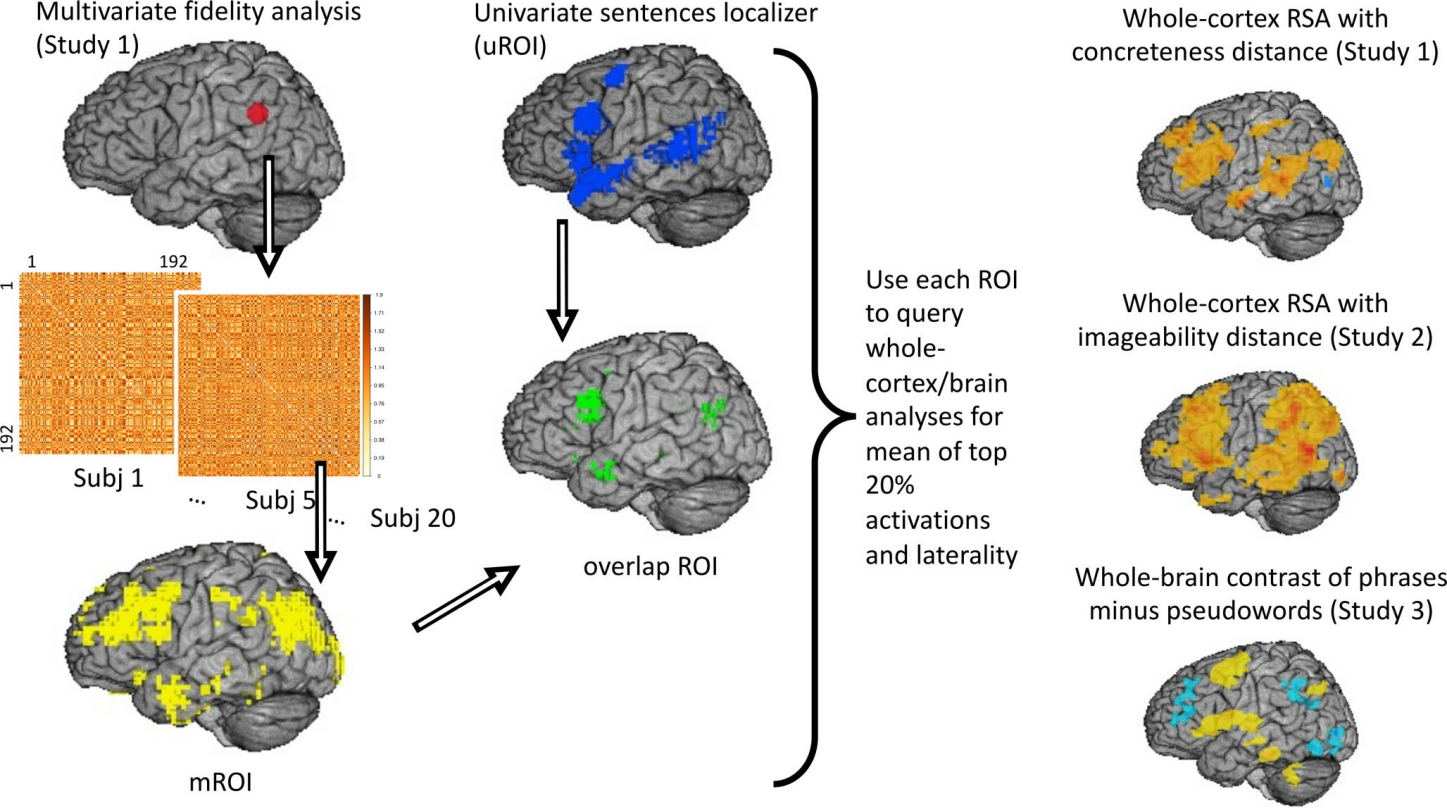
Overview of the study methods. Representational fidelity analysis was used to define multivariate regions of interest (mROI). An external localizer based on a contrast of sentences > pseudowords was taken from existing studies (Fedorenko et al. 2010) and used as the univariate region of interest (uROI). The spatial overlap between the mROI and uROI formed the third ROI. These were then used to query whole-cortex results from RSA analyses of single-word semantics, and whole-brain results from a univariate contrast of multi-word phrases compared to pseudowords

Due to the largely methodological nature of this study, we first outline the overall approach (Fig. 1) before providing additional details below. The approach is developed in our primary study (Study 1), then validated on two independent datasets (Studies 2 and 3). Starting with Study 1, to compare multivariate with univariate approaches to localizing language cortex, we first created a set of multivariate regions of interest. This was done using representational fidelity (RF) analysis (Rothlein et al. 2018). We wanted to be inclusive at this stage, so we based the RF analysis on data from visual word presentations relative to an implicit (visual fixation) baseline. This resulted in a map of voxels showing consistent responses across words and subjects, which we used as the multivariate regions of interest (mROI). We then compared this with a widely used set of univariate regions of interest (uROI) for language, developed and made available by Fedorenko et al. (2010).

The uROI were based on a contrast of sentences > pseudowords, the results of which are thought to highlight neural areas processing semantics and syntax. To facilitate as direct a comparison as possible with the univariate approach while maintaining the use of simple stimuli on which we can exert tight experimental control, we focused on single-word semantics. Our primary analyses involved RSA (Kriegeskorte et al. 2008), in which the predicted Representational Dissimilarity Matrices (RDM) were defined in terms of differences in semantic measures among stimuli. These predicted RDM were then compared to observed RDM defined in terms of neural responses to each stimulus within a searchlight. To account for properties of words other than semantics, analyses were conducted in terms of partial correlations that also included predicted RDM for orthographic and phonological word properties. Resulting parameter estimates were queried and compared between the mROI, uROI, and their spatial overlap. Lateralization indices were also calculated as a measure of external validity.

The mROI was defined in terms of words compared to baseline, which is independent of the predicted RDM for words defined in terms of semantics. However, the surest test of independence is to apply the mROI to different data. That is what we did in Studies 2 and 3. In Study 2, the predicted RDM was again defined in terms of semantics. In Study 3, we used a dataset that might be expected to favor the uROI approach. The stimuli were multi-word phrases, and activations were defined in terms of univariate contrasts. In all three studies, we compared activation and laterality indices for mROI, uROI, and their spatial overlap (Fig. 1).

### Study 1 (primary experiment)

#### Participants

We recruited 20 neurotypical, right-handed speakers of English as a first language who were between the ages of 18 and 24 (13 female, 7 male). Mean age was 20 (SD: 1.54) years. Participants were recruited from the Rutgers University-Newark campus and completed an online screening form to assess eligibility and MRI safety. From the screening responses, eligibility was determined by absence of (1) any history of neuropsychological disorders (past or present), (2) psychoactive medication use or drug/alcohol abuse, (3) left-handedness, (4) English learned after five years old, (5) history of medical conditions that indicate neurological or physiological disturbance (e.g., severe concussion, diabetes, fainting spells), and (6) presence of metal in soft body tissue not anchored in bone. Participants provided written informed consent in accordance with Rutgers University Institutional Review Board protocol.

#### Stimuli

The 192 total words in the stimulus set consisted of 128 abstract and 64 concrete words. Twice as many abstract than concrete words were included because of a separate planned analysis to compare abstract words based on internal features (e.g., emotion, thought, morality) versus external features (e.g., time, space, number). Because that analysis is not relevant to the current study, we considered all 128 abstract words together. Characteristics were compared between abstract and concrete words using standard two-sample *t*-tests. The abstract words differed significantly from the concrete words on rated concreteness, based on a large independent set of ratings (Brysbaert et al. 2014), but were otherwise matched (not significantly different) on word frequency (log_10_-transformed occurrences per-million values), orthographic length (number of letters), number of syllables, orthographic Levenshtein distance (OLD20, average distance between a word and its 20 nearest orthographic neighbors; Yarkoni et al. 2008) and bigram frequency (log_10_-transformed per-million values for words that share the same two-letter pair in the same position as the target word). Word frequency estimates were obtained from the SUBTLEX-US database (Brysbaert and New 2009), number of syllables and Levenshtein distances were computed using the quanteda and vwr packages in R, respectively (Benoit et al. 2018; Keuleers 2013), and bigram frequencies were obtained from the McWord online database of calculations based on CELEX (Baayen et al. 1995; Medler and Binder 2005). See Table 1 for summary of word characteristics.

**Table 1:** Characteristics of the word stimuli. Abstract and concrete words did not reliably differ (*p* > = 0.1) across any listed condition except the target factor of concreteness (*p* < 0.001). Values for abstract and concrete words are given as means (standard deviations, SD)

Word characteristic	Abstract	Concrete	*t*(190)
Concreteness rating (1–5, low-high concreteness)	2.45 (0.59)	4.79 (0.19)	30.91
Word frequency (log_10_)	6.20 (1.87)	6.03 (1.44)	0.63
Bigram frequency (log_10_)	6.44 (0.93)	6.24 (0.89)	1.44
Length (letters)	7.45 (2.53)	7.59 (1.60)	0.41
Syllables	2.59 (1.13)	2.45 (0.71)	0.91
OLD20	2.44 (0.75)	2.64 (0.85)	1.69

#### Task

During fMRI, participants performed a familiarity judgment task where they indicated with a button-press whether or not the word presented on the screen is one that they use or hear often (is familiar) or do not use or hear often (is unfamiliar). This task was adapted from Wang et al. (2018) and chosen (1) to encourage participants to focus on each word, up to and including its meaning, while avoiding undue engagement of additional processes such as working memory or meta-cognitive evaluation, and (2) to elicit measurable responses so as to ensure continual task engagement.

PsychoPy software was used for stimulus delivery and response collection (Peirce 2007). Participants were given an MRI-compatible two-button box and instructed to press one button if the word was one that they use or hear others use often (familiar), and the other button if the word was one that they do not use or hear others use often (unfamiliar). An initial practice condition was included that provided examples of words that might be used or heard often (e.g., water) and words that might not be used or heard often (e.g., veal) for additional clarity. The experiment followed a randomized, event-related design. Following a similar paradigm to Wang et al. (2018), each trial consisted of the following: First, a fixation cross was presented in the middle of the screen for 500 ms, then the stimulus (word) was displayed for 1500 ms, and then another fixation cross was displayed for 500 ms. Then, the screen returned to a fixation cross for an inter-trial interval (ITI, jitter), randomly jittered for ≥ 2000 ms. Variable ITIs were created by randomly interspersing 96 null trials (a trial in which a fixation is displayed and no task is required) in each run such that the minimum 2000 ms fixation following the word trial would be extended by the number of any null events. Four null trials (8 s of fixation) were also included at the beginning and end of each run. Reaction time was recorded at the time of the first button press after stimulus onset.

All 192 unique word trials were fully randomized across all conditions and arranged into two “runs” (uninterrupted sets of trials with continuous image acquisition), with 96 words per run. Following these initial two runs, each word appeared twice more in subsequent runs, for a total of three times across the six runs in the experiment. Words spanned a range of frequencies (an indirect measure of familiarity) to keep participants engaged throughout the task (log_10_-transformed word frequency min = 1.79, max = 11.79).

#### MRI data acquisition and processing

Structural and functional brain data were acquired using a Siemens Trio 3 Tesla MRI scanner (Erlangen, Germany) with a 12-channel head coil at the Rutgers University Brain Imaging Center. T1-weighted (1 mm isotropic resolution) structural images were obtained using a Magnetization Prepared Rapid Gradient Echo (MPRAGE) sequence (TR = 1900 ms, TE: 2.52 ms, matrix = 256 × 256 voxels, 176 contiguous axial slices, field of view (FOV) = 256 mm). T2*-weighted (3 mm isotropic resolution) Blood Oxygen Level Dependent (BOLD) functional image slices were acquired in an interleaved order using a gradient-echo echoplanar imaging (EPI) sequence (TR = 2000 ms, TE = 25 ms, matrix = 64 × 64, 35 axial slices, FOV = 192 mm). Two hundred whole-brain volumes, each consisting of 35 axial slices, were acquired for each of the six runs.

Analysis of Functional NeuroImages (AFNI) software (Cox 1996) and the FMRIB Software Library (FSL; Jenkinson et al. 2012) were used to preprocess neuroimaging data. Specifically, pre-processing steps prior to multivariate analysis consisted of the following: Motion correction and slice-timing correction using the AFNI script, align_epi_anat.py (Saad et al. 2009). Each of the six functional runs was aligned within-runs to the mean image, then the runs were aligned to each other with the third run as the target. For slice-timing correction, the first four time points, during which no task occurred, were ignored to avoid potential image saturation effects. The motion-corrected and slice-timing corrected runs were then concatenated together as input to the AFNI program 3dDeconvolve to generate the full design matrix. Also included as inputs to 3dDeconvolve were an inclusive mask for the EPI data, a censor for the first four TRs, and seven nuisance covariates (covariates of no interest): Six motion parameters (one each for rotation and displacement in the pitch, roll, and yaw directions), and the first principal component of signal from the lateral ventricles, as segmented using the FSL automated segmentation tool, FAST (Zhang et al. 2001). The resulting design matrix and concatenated functional runs were then input to the AFNI program 3dLSS, which uses the least-squares-sum regression approach described by Mumford et al. (2012) to derive beta-weight images for each stimulus trial. These beta-weight images were re-ordered such that the images corresponding to the stimulus responses were placed in the same order for all participants. This allowed for the same representational dissimilarity matrices (RDM) to be used for each participant. The resulting images were then aligned to a common group space (Talairach space; Lancaster et al. 2000) using nonlinear diffeomorphic routines as implemented in the AFNI script, @SSwarper. Those images served as inputs for all subsequent multivariate analyses.

#### Representational fidelity analysis and multivariate localizer

The multivariate region of interest (mROI) was defined using pattern-based fidelity analyses, in which the basic elements of the analyses were the RDM. Fidelity analyses, as a measure of reproducibility, were performed with these RDM using leave-one-out cross-validation (as in Rothlein et al. 2018). Here the 20 participants read 192 words that were presented 3 times each. An observed RDM was constructed as a word by word matrix containing all the words (as shown on the left side of Fig. 1), where the elements being compared for each word comprised a vector of activations in a searchlight. The activations in the vector reflect responses to words (averaged over the 3 presentations of each word) compared to a fixation baseline, without regard to particular properties of the words. The searchlight was a sphere with a radius of 3.5 voxels, containing 123 total voxels. Representational Fidelity (RF) is computed within each searchlight by taking all the RDM (1 matrix consisting of an average across the 3 occurrences of each word x 20 participants = 20 RDM) and computing the leave-one-RDM-out reliability: correlate (RDM1, mean (RDM2 through RDM20). RF for RDM1 is the resulting correlation coefficient. This result was calculated for each voxel in the searchlight. The searchlight sphere was moved over the whole cortex, such that each gray matter voxel served as its center exactly once. This analysis results in a whole-cortex map highlighting the areas showing consistent multivariate patterns of the multiple word presentations across subjects. The resulting mROI can subsequently be used to focus analyses based on predicted RDM defined in terms of, for example, word-word differences in concreteness, imageability, or other relevant measures (right side of Fig. 1). To ensure an inclusive mROI, the RF results were thresholded at a voxel-level *p* < 0.05. An extent threshold of 120 voxels was also applied. This threshold was derived following the recommendations of Nieto-Castañón and Fedorenko (2012) for applying a relaxed threshold at the voxel level to make an inclusive mask. Because this resulted in some extra-parenchymal voxels that were quite unlikely to be physiologically relevant, an extent threshold of 120 was applied that minimized such voxels while maintaining voxels in relevant areas such as the ventral temporal lobe.

#### Univariate localizer

For comparison with the multivariate localizer defined based on data from Study 1, we used a univariate localizer (uROI) based on separate data. This was adopted from Fedorenko et al. (2010), with the only change being that a nonlinear warp, calculated using the AFNI script @SSwarper as described above, was applied to move the uROI into Talairach space (Lancaster et al. 2000). The Fedorenko et al. localizer is based on the contrast of sentences > pseudowords (made available at https://evlab.mit.edu/funcloc/). Areas highlighted by this uROI are qualitatively distinct from the mROI in that it reliably engages superior and middle temporal gyri (outlined in white in Figs. 3 and 4).

#### Predicted representation matrices

The primary relationship of interest among the word stimuli was in terms of their semantics. The predicted semantic RDM was defined in terms of differences in concreteness for each word pair, where each word has a rated concreteness value (Brysbaert et al. 2014). The stimulus-stimulus distance matrix was defined as the absolute value of the difference in concreteness between each pair of words in the stimulus set. Stimulus-stimulus distance matrices defined in terms of phonological and orthographic edit distance measures were used to partial out effects of phonology and orthography. As expected, semantic distances were not significantly correlated with orthographic or phonological distances (|*r*| < 0.02, *p* > 0.05).

Word dissimilarities for phonology and orthography were defined in terms of their pair-wise distance as the number of edits needed to make the pair identical. To give an orthographic example, bullet and wallet have an edit distance of 2 because only “bu” and “wa” differ between them. However, wallet and jacket have an edit distance of 3 because “wal” and “jac” all differ between that pair. Phonological edit distance is defined similarly, except that phonemes are used instead of letters, and phonetic features of place and manner of articulation are also taken into account when determining whether two phonemes of a word are identical (Hall et al. 2019). Including phonetic features when calculating phonological edit distance attenuates the correlation between representations defined in terms of orthography and phonology such that orthographic and phonological distances for the current set of word stimuli are only correlated at *r* = 0.34. This modest level of correlation allows them to be included in the same partial correlation analysis, as we have done previously for other word stimuli (Graves et al. 2023).

#### Representational similarity analyses (RSA)

RSA compares the predicted RDM to the observed (neural) RDM. For this Study 1 RSA, the same neural data were used as for the RF analyses discussed above. This is justified because the RSA and RF analyses are orthogonal to each other. Whereas the RF analyses are based on the correlations among observed RDMs across participants, the RSA analyses are based on comparing predicted to observed RDMs within participants. Still, there may be concerns about independence. We therefore also include an analysis with independent datasets (see Study 2 analysis below).

To test for differences in *sensitivity* between the mROI and uROI in the case of multivariate analysis, we compared mean parameter estimates (beta weights for partial correlations in RSA) extracted from within the mROI, uROI, and their spatial overlap. The partial correlation RSA was conducted as a whole-cortex searchlight to test for brain areas related to semantic representations, as distinct from orthographic and phonological representations. This was done using CoSMoMVPA software (Oosterhof et al. 2016). The observed RDM were based on vectors of neural signal intensity (beta weights). Beta values were z-score normalized across stimuli within each voxel. The observed (neural) and predicted RDMs were then compared using Spearman’s rho, and the resultant value was assigned to the center voxel of the searchlight. The searchlight was moved over the whole cortex, such that each gray matter voxel served as its center exactly once. The resulting correlation coefficient maps for each subject were then smoothed using a 5 mm full-width half-maximum kernel and entered into a 1-sample t test, before being Fisher z-transformed and thresholded at a voxel-level *p* < 0.005 and map-wise cluster corrected to *p* < 0.05. In this and subsequent studies, when querying all the ROI we aimed for stability of signal and comparability across ROI by taking only the top 20% most active voxels, as established previously (Mitsis et al. 2008). That is, comparisons among the mROI, uROI, and their overlap were carried out as comparisons among the top 20% most active voxels in each case.

Additionally, in a supplementary analysis we checked to see if the across-subjects measure of representational fidelity constituting the mROI was potentially conflated with neural responses to differences in how familiar subjects judged the words to be according to the task. Words judged to be familiar were coded with a 1, and unfamiliar with a 0. These values were averaged over the three instances in which the word appeared, and mean familiarity was compared pair-wise by taking the absolute value of the difference between each word pair. Those values made up the RDM for a searchlight RSA analysis, performed as described for the other searchlight RSAs above. Note that this method entailed having a different RDM for each subject, reflecting each subject’s pattern of familiarity judgments across the word stimuli.

To test for differences in *validity* between the mROI and uROI, we followed the logic outlined by Wilson et al. (2017). The left hemisphere is known to house the majority of critical cortex for language in neurotypical participants, so a more positive laterality index (LI) indicating left-lateralization is indicative of greater face validity of the results. We used the standard formula (Binder et al. 1996; Desmond et al. 1995): LI = (V_Left_ – V_Right_)/(V_Left_ + V_Right_), where in this case V is the number of significant voxels within the localizer in the given hemisphere.

### Study 2

To insure against the possibility that defining the mROI using the same data as subsequent RSA analysis (albeit for the independent conditions of words compared to fixation for the mROI, and correlations with predicted RDM for RSA analysis) might lead to over-fitting or a degree of logical circularity (Kriegeskorte et al. 2009), we performed similar analyses to Study 1 in an independent data set. In Study 2, participants made lexical decisions to visually presented words. The nonword foils were pseudowords. These foils were chosen so that lexical decisions would be based primarily on whether the letter string was meaningful (a semantic criterion), as opposed to simply pronounceable (a phonological criterion) or visually familiar (an orthographic criterion). This dataset was published previously and is more extensively documented in Graves et al. (2017). A brief description of the most relevant elements follows.

#### Participants, task, and stimuli

A total of 20 participants (13 women, 7 men), all right-handed with English as a first language and reporting no neurological or psychiatric diagnoses or history of learning disability, gave written informed consent to participate in the study. Their mean age was 25.3 years, with 16.6 mean years of education. During fMRI scanning, participants performed a visual lexical decision task, in which participants indicated with a button press whether or not they judged the string of letters being displayed to form a valid English word. A total of 312 words and 312 pseudowords were randomly intermixed and presented across 6 runs in the experiment. The words were selected to be of either high or low frequency and high or low imageability, in a completely crossed 2 × 2 factorial design. Pseudowords were generated to contain valid English trigram (3-letter) sequences to ensure pronounceability. They did not significantly differ from words in terms of number of letters, bigram frequency, or trigram frequency.

#### MRI data acquisition and processing

MRI data were acquired using a 3T GE Excite system with an 8-channel array head coil. Acquisition parameters were as follows: To ensure high quality anatomical images, we acquired two T1-weighted high-resolution anatomical images, one in axial orientation with a resolution of 0.938 × 0.938 × 1.000 mm, and one in sagittal orientation (1.000 × 0.938 × 0.938 mm), each consisting of 180 contiguous slices. Functional EPI scans were acquired with 25 ms TE, 2000 ms TR, 208 mm FOV, 64 * 64 pixel matrix, in-plane voxel dimensions of 3.25 × 3.25 mm, and slice thickness of 3.3 mm with no gap. The 41 axial slices were acquired in interleaved order, and each of the 6 functional runs consisted of 140 whole-brain volumes.

The MRI data were pre-processed as described in Graves et al. (2017), including field unwarping, slice-timing correction, and motion correction. Beta-weight images were then derived for each stimulus trial using least-squares-sum regression (Mumford et al. 2012), implemented in the AFNI program, 3dLSS as described above for Study 1.

#### Representational similarity analyses

We performed RSA on this dataset, where the predicted RDM of interest was defined in terms of imageability, a measure of the subjective degree to which a word calls to mind a sensory impression. This measure of single-word semantics has been shown to be highly correlated with concreteness (Altarriba et al. 1999). The predicted orthographic and phonological RDM were defined and calculated as described for Study 1, but for the distinct stimuli in Study 2. For the word stimuli in this dataset, the orthographic edit distance and the phonological edit distance are correlated for the set of words at *r* = 0.46 (*p* < 0.001). However, levels of multi-collinearity below *r* = 0.7 are generally considered to not violate the assumptions of the general linear model, of which partial correlation analyses are a special case (Kutner et al. 2005).

### Study 3

An additional study was included to test the possibility that the univariate localizer would be better suited for detecting activation from univariate contrasts. Additionally, the uROI localizer based on multi-word combinations may be a better fit to data from participants tested using multi-word (in this case, article-noun-noun) combinations, whereas the mROI localizer based on single-word data may be a better fit for testing experiments using single-word stimuli. Note that the mROI and uROI used to query results (averaging activations across each voxel in the ROI) are identical to the ones used in Studies 1 above and – as in Study 2 – are defined independently of the current dataset. This dataset was published previously and is more extensively documented in Graves et al. (2010a, b). A brief description of the most relevant elements follows.

#### Participants, task, and stimuli

A total of 22 participants, all right-handed with English as a first language and reporting no neurological or psychiatric diagnoses, gave written informed consent to participate in the study. Their mean age was 24.7 (SD: 5.4), with 15 females and 7 males. During fMRI scanning, participants were asked to press one button if the phrase displayed was meaningful, another if not meaningful, and a third if it was made of pseudowords. The noun-noun phrases were presented in either sensible order, e.g., the ski jacket, or reversed order, e.g., the jacket ski. They were taken from a larger human-rated set (Graves et al. 2013), and selected for being maximally sensible in forward but minimally sensible in reversed order. Pseudoword phrases, e.g., the rola brip, were presented as a comparison condition. The pseudowords were matched to words on the surface characteristics of length (in total number of letters) and bigram frequency (a measure of orthotactic typicality), as retrieved from MCWord (Medler and Binder 2005). Participants were shown a total of 200 forward (meaningful) phrases, 200 reversed (non-meaningful) phrases, and 200 pseudoword phrases.

#### MRI data acquisition and processing

The MRI data were acquired using a 3T GE Excite scanner with an 8-channel array head coil and the following parameters: T1-weighted high-resolution anatomical images had a resolution of 0.938 × 0.938 × 1.000 mm across 134 contiguous axial slices. Functional EPI scans were acquired with 25 ms TE, 2000 ms TR, 224 mm FOV, 64 * 64 pixel matrix, in-plane voxel dimensions of 3.5 × 3.5 mm, and slice thickness of 3.0 mm with a 0.5 mm gap. The 33 axial slices were acquired in interleaved order, and each of the 4 functional runs consisted of 232 whole-brain volumes.

Subsequent processing steps were as described in Graves et al. (2010a, b), including smoothing at 5 mm FWHM and thresholding at a cluster-corrected *p* < 0.05, applied to the contrast of meaningful phrases minus pseudoword phrases. Volumetric results were then mapped onto the nearest gray matter surface for display (Fig. 4) using the AFNI program 3dVol2Surf, and rendered using SUMA software (Saad and Reynolds 2012).

## Results

### Study 1 (Primary Experiment)

**Fig. 2:**
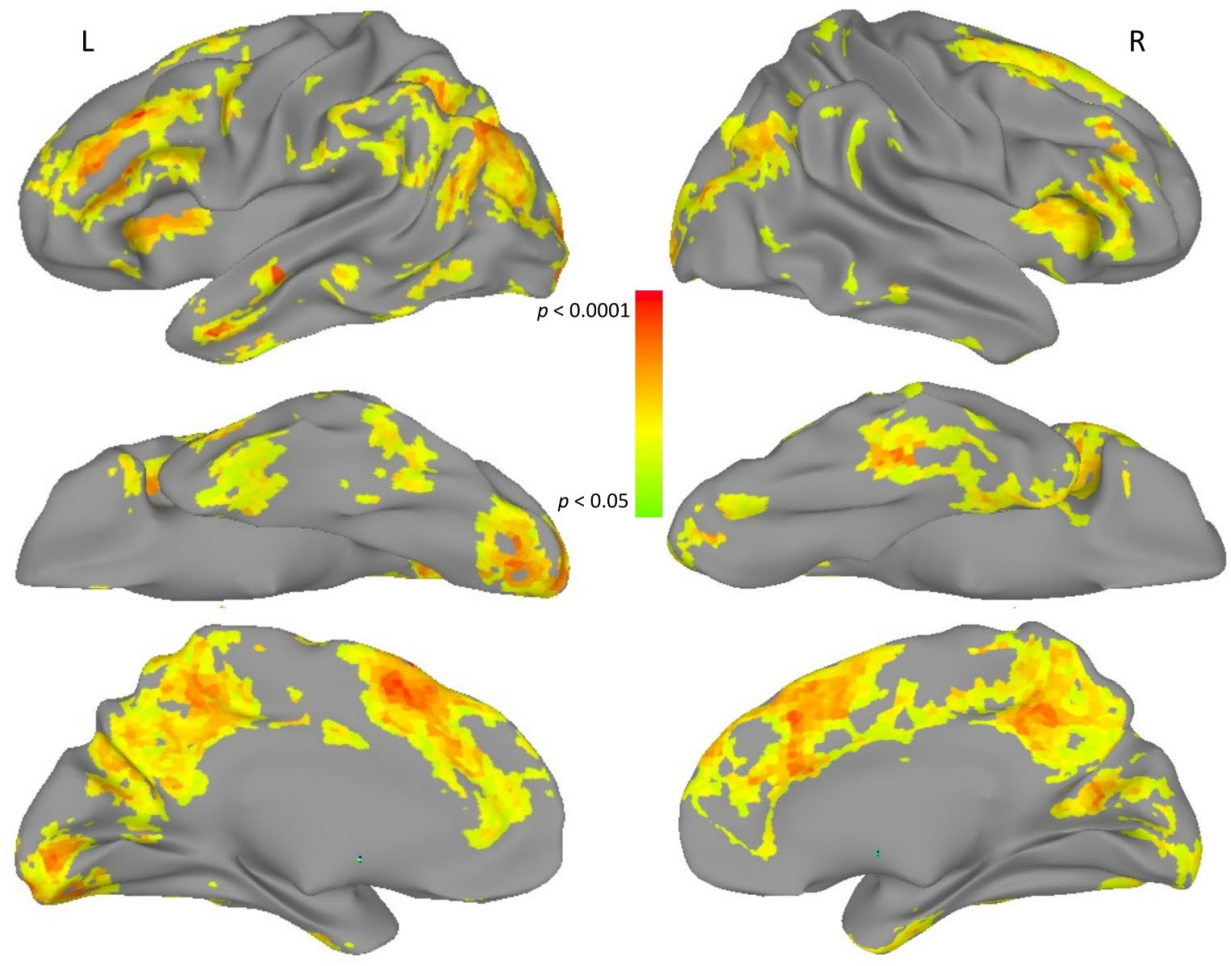
Representational fidelity results, thresholded and projected onto the nearest cortical surface for both hemispheres

Results of the representational fidelity analysis are shown projected onto the nearest cortical surface in Fig. 2, with labeled coordinates of local peaks in Talairach space in Table 2. Note that to provide additional detail for larger clusters in the coordinate tables, we list local maxima within them that have a separation distance of at least 11 mm, as derived using the AFNI program, 3dExtrema. The fidelity analysis resulted in an mROI that included both peri-Sylvian and extra-Sylvian association cortices, as well as sensory and motor regions.

**Table 2:** Results of the representational Fidelity analysis defining the multivariate regions of interest. Peak coordinates are labeled by the landmark-based atlas structure in which they fall, along with the size of the overall cluster within which they appear and the corresponding z-score magnitude. R: right, L: left, SMA: supplementary motor area

Location of extreme point	Cluster size (mm^3^)	X	Y	Z	z-score
Bilateral superior frontal gyrus	6221	23	-8	66	5.55
R medial superior frontal gyrus/SMA		2	20	48	4.38
R cuneus		14	-62	18	4.26
R medial superior frontal gyrus/pre-SMA		2	26	36	3.96
R mid-cingulate cortex		2	-5	39	3.72
R medial superior frontal gyrus		2	38	45	3.53
L anterior cingulate cortex		-2	38	6	3.13
R anterior cingulate cortex		5	44	15	2.92
R middle frontal gyrus	449	29	32	27	4.14
L anterior middle temporal gyrus	296	-56	2	-22	4.77
L anterior fusiform gyrus		-32	-14	-25	2.91
R fusiform gyrus	268	44	-29	-25	4.55
L anterior insula	152	-32	20	12	4.09
L fusiform gyrus	120	-47	-44	-16	3.27

**Fig. 3:**
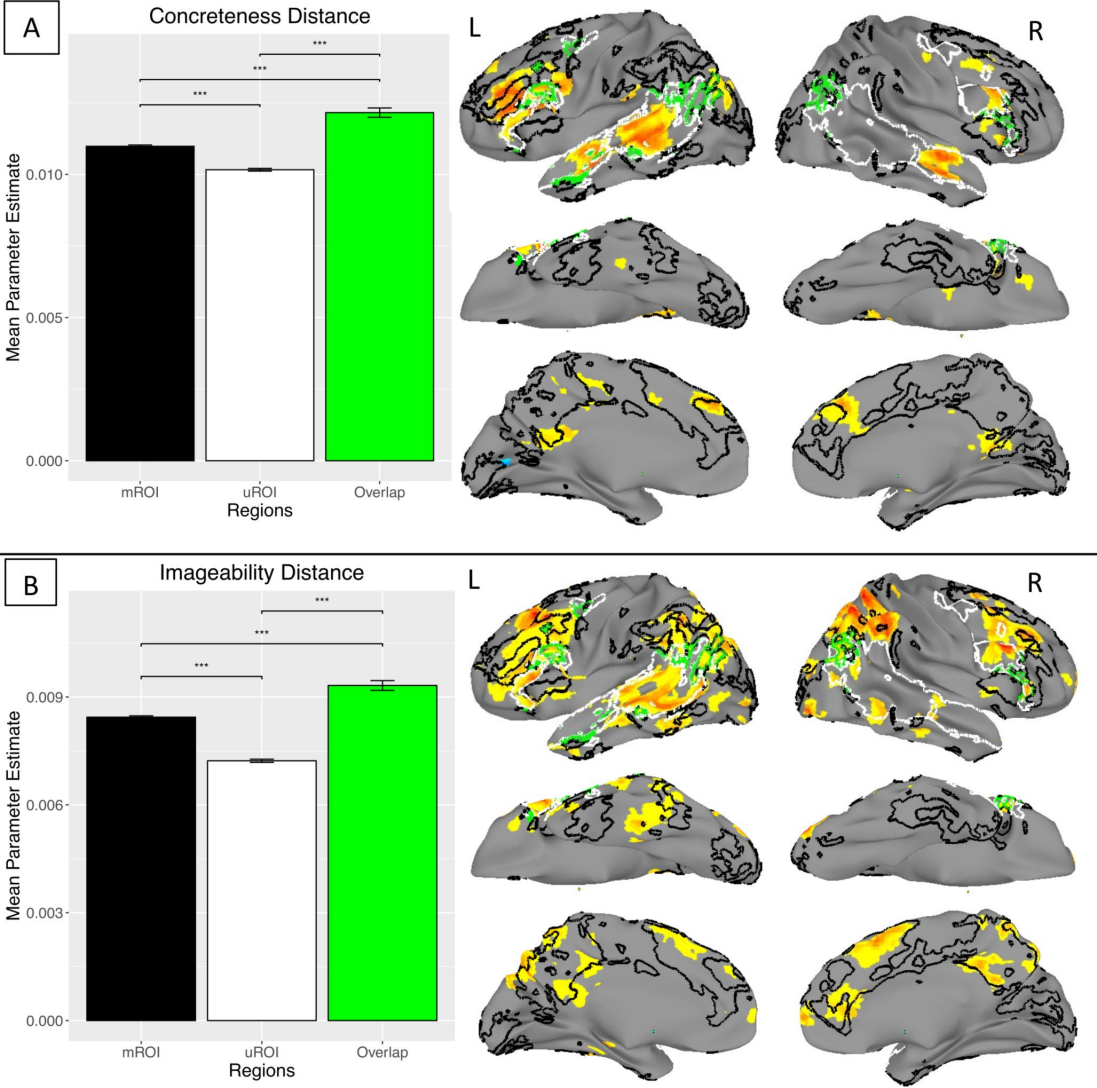
Results from partial correlation RSA, based on (**A**) concreteness distance from Study 1, and (**B**) imageability distance from Study 2. Note that areas in the mROI (black outlines and bars) show greater correlation with the RDM than the uROI (white outlines and bars). Areas of overlap between ROI (green outlines and bars) show the greatest activation. *** *p* < 0.001

**Fig. 4:**
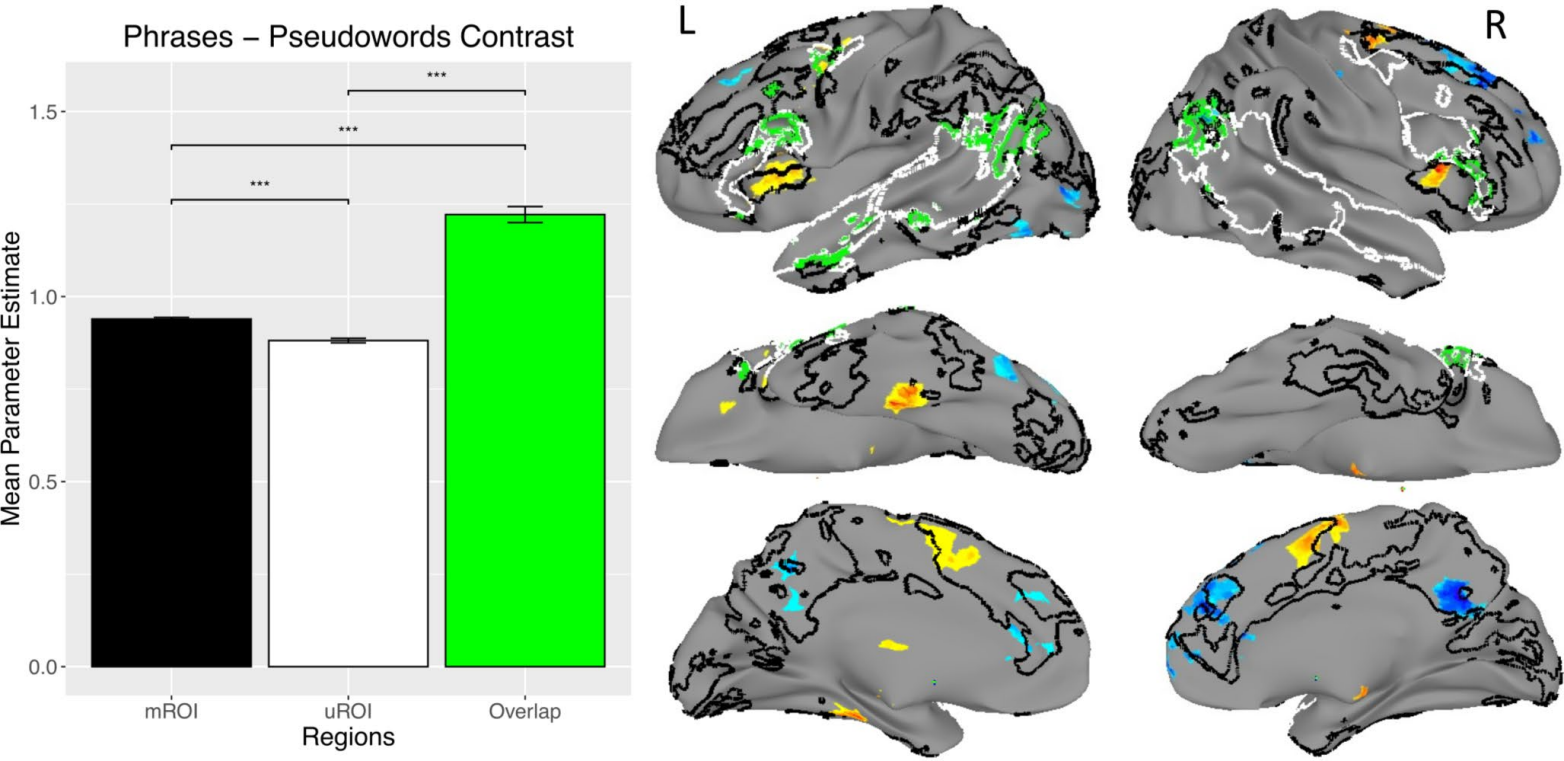
Results from a univariate contrast of meaningful noun-noun phrases (hot colors) with pseudowords (cool colors). Greater activations are found within the mROI than the uROI. Areas of overlap between ROI show the greatest activation in all cases. *** *p* < 0.001

The mROI localizer areas shown in Fig. 2 are outlined in black in Figs. 3 and 4. The mROI are derived from the Study 1 data, while the univariate localizer areas (uROI), outlined in white in Figs. 3 and 4, are from analyses of other datasets (Fedorenko et al. 2010). Areas where the mROI and uROI spatially overlapped are outlined in green.

Recall that the mROI was defined by representational fidelity analysis, such that areas within the mROI reflect voxels that show high reproducibility of multivoxel patterns across runs and subjects. The uROI, on the other hand, reflects areas defined by Fedorenko et al. (2010) on different datasets, using a univariate contrast of sentences > pseudowords. Our hypothesis that the mROI would be more inclusive than the uROI was supported by the fact that the mROI showed a greater spatial extent (5,546 voxels) than the uROI (3,338 voxels), where each voxel was 3 mm^3^ isotropic. Note that their amount of spatial overlap was relatively small at 449 voxels (cf. black, white, and green outlines in Fig. 3). Separated out by hemisphere, the size of the mROI-uROI overlap in the left hemisphere was 307 voxels, while in the right it was 142 voxels.

To test the hypothesis that the mROI would be more sensitive to neural associations with language representations, we chose to focus on semantic representations in the RSA analysis, with orthographic and phonological representations partialed out, as this was assumed to be most similar to the results of the sentences > pseudowords contrast from which the uROI was defined. Taking the mean of the top 20% of the voxels in the mROI, the uROI, and their spatial overlap, we performed two-tailed *t*-tests to compare mean parameter estimates. Results of that analysis are shown for Study 1 in Fig. 3A, with peak coordinates listed in Table 3.^1^

**Table 3:** Results of the representational Similarity Analysis whole-cortex searchlight, where the predicted RDM was defined in terms of either differences in word concreteness (Study 1) or imageability (Study 2). Peak coordinates are labeled by the landmark-based atlas structure in which they fall, along with the size of the overall cluster within which they appear and the corresponding z-score magnitude. L: left, R: right, SMA: supplementary motor area

Location of extreme point	Cluster size (mm^3^)	X	Y	Z	z-score
*Concreteness (Study 1)*					
L inferior frontal gyrus, pars triangularis	459	-47	17	18	4.42
L posterior superior temporal sulcus	260	-59	-38	6	4.01
R medial superior frontal gyrus	173	5	32	39	3.94
L anterior fusiform gyrus		-32	-14	-25	2.91
R inferior frontal gyrus, pars triangularis	161	47	17	27	3.70
Bilateral posterior cingulate	152	-11	-56	21	3.34
R posterior cingulate		11	-47	15	3.32
L angular gyrus	136	-26	-71	27	3.71
R superior temporal gyrus	107	53	-8	-10	4.12
*Imageability (Study 2)*					
Bilateral temporo-parietal cortex	2642	-53	-65	12	4.67
R posterior cingulate		2	-47	30	3.69
L middle frontal gyrus	1098	-35	23	39	4.68
R middle frontal gyrus	444	32	8	54	4.33
R SMA	265	8	14	51	3.82
R pre-SMA		5	29	48	3.37
L anterior cingulate		-2	23	30	2.92

Areas showing significant neural associations with concreteness from the whole-cortex searchlight RSA included the bilateral middle frontal, inferior frontal, and medial superior frontal gyri, bilateral anterior superior temporal gyrus, bilateral posterior cingulate cortex, left middle temporal gyrus, and the left angular gyrus. Activation in the middle and inferior frontal gyri was of greater spatial extent on the left than right. Mean differences among the ROI are shown in the bar graphs (Fig. 3A), with the mROI showing a significantly greater mean RSA Parameter Estimate than the uROI, and their overlap showing greater activation than either alone.

A supplementary analysis was performed to determine the extent to which the representational fidelity comprising the mROI might be influenced by differences in how familiar participants judged the words to be. We calculated word-word familiarity distance matrices for each subject based on their own familiarity judgments. These showed minimal Spearman (rho) correlation with the RDM based on word-word concreteness (mean: 0.05, min: 0.01, max: 0.16). We also performed a whole-cortex RSA searchlight for areas showing correspondence between familiarity judgments and concreteness. As shown in Supplementary Figure (SF) 1, this analysis revealed largely dorsal stream areas, more on the left than the right, to be associated with patterns of familiarity judgments, as calculated separately for each subject. The representational fidelity results comprising the mROI, by contrast, were largely associated with ventral stream areas. Areas of spatial overlap between the two results were also partially left-lateralized, largely appearing in the left lateral parietal lobe, bilaterally in the anterior insula and lateral frontal lobe, and medially in the supplementary motor area (SMA) and pre-SMA. Note that the areas of overlap represented only a subset of the areas comprising the mROI, with almost no overlap occurring in the ventral temporal and occipital cortices.

For comparison, we also tested for areas of overlap between the subject-specific familiarity RSA and the uROI. Qualitatively, many of the areas showing overlap between the familiarity task results and the uROI were also areas that overlapped between the mROI and uROI, including the left anterior temporal lobe, posterior superior temporal gyrus, angular gyrus, and inferior frontal gyrus (SF 2).

### Study 2

To ensure that the results from Study 1 were not specific to the task, or simply due to the fact that the mROI was defined based on the same dataset that the semantics-focused RSA was performed on, we applied the same ROIs to data from two additional studies using different tasks. In the first of these, Study 2, words were not repeated, the task was lexical decision rather than familiarity judgment, and semantics was operationalized in terms of imageability rather than concreteness. The use of somewhat older imageability norms, as complied from multiple sources for use in fMRI analysis by Graves et al. (2010a, b) and made freely available through the SCOPE database (Gao et al. 2022), reflects not a theoretical choice but rather the fact that Study 2 was a re-analysis of legacy data. However, imageability and concreteness have been shown to be highly correlated (*r* = 0.87), suggesting that both measures operationalize the same semantic factor (Altarriba et al. 1999).

Results of the whole-cortex RSA searchlight showed neural associations with imageability in bilateral medial superior, middle, and inferior frontal gyri (greater on the left than right), bilateral inferior parietal lobule and posterior cingulate cortex, a largely left-lateralized swathe of superior and middle temporal gyri, right anterior cingulate cortex, and left temporo-occipital (fusiform) gyrus (Fig. 3B, coordinates in Table 3). Considering the mean of these results across voxels within each ROI, the mROI again showed significantly greater neural association with imageability compared to the uROI, with the spatial overlap of the two showing greater correlation with the RDM than either alone.

### Study 3

In the previous experiments, Study 1 defined the mROI and tested them against the uROI for neural sensitivity to semantics in an experiment using single-word presentation. Study 2 took a similar approach to comparing the ROI but did so in an independent dataset. In Study 3, we tested the possibility that the mROI might be sensitive to neural correspondence with single-word semantics, but the uROI might be more sensitive to multi-word semantics. Additionally, the activations within the ROIs in Study 3 were from a univariate contrast of meaningful article-noun-noun phrases compared to length-matched article-pseudoword-pseudoword stimuli. This allowed us to test the possibility that the uROI (defined by an independent univariate contrast) would be more sensitive than the mROI (defined by multivariate representational fidelity analysis) to activation from univariate contrasts.

Because the task in this study likely involved two cognitive steps for the phrases: (1) recognizing that the phrase was made of real words, and then (2) determining phrase-level meaning (compared to only requiring step 1 for pseudowords), we also report the behavioral results for the two conditions. Considering all trials, means were compared in terms of accuracies and response times and showed complementary results. Pseudowords were correctly identified more often (88.6%, SD: 45.4) than meaningful phrases (85.3%, SD: 35.4), and this difference was significant (*t* = 3.76, *p* < 0.001). Likewise, response times from stimulus onset to button press were faster for pseudowords (mean: 856.2 ms, SD: 257.8) than for meaningful phrases (951.3 ms, SD: 297.2), and this difference was significant (*t* = 16.2, *p* < 0.001).

As shown in Fig. 4 (and listed in Table 4), activations for meaningful phrases relative to pseudowords occurred primarily in bilateral inferior frontal cortex, supplementary motor area, and left parahippocampal gyrus. The comparison contrast of activation for pseudoword phrases relative to meaningful phrases occurred primarily in bilateral dorso-medial prefrontal cortex and posterior cingulate, greater on the right than left. However, pseudoword-related activations were not of interest for the current study and did not enter into the current analysis due to our focus on the top 20% most active voxels in each ROI. As in the previous studies, activations in the mROI were significantly greater than those in the uROI, with their spatial overlap showing the greatest level of activation.

**Table 4:** Results of the univariate contrast of meaningful phrases minus pseudowords (Study 3). Peak coordinates are labeled by the landmark-based atlas structure in which they fall, along with the size of the overall cluster within which they appear and the corresponding z-score magnitude. R: right, L: left, SMA: supplementary motor area

Location of extreme point	Cluster size (mm^3^)	X	Y	Z	z-score
*Phrases > pseudowords*					
Bilateral SMA	6193	0	0	55	4.98
L caudate	4508	-10	-1	15	5.37
L anterior insula	3034	-31	10	12	4.85
R caudate	1957	11	6	15	5.30
L posterior middle frontal gyrus	1896	-35	-3	53	4.50
L fusiform gyrus	1506	-27	-27	-17	6.56
Medial cerebellum	1405	1	-52	-29	5.14
R posterior middle frontal gyrus	1197	23	-12	51	4.96
R lateral cerebellum	1118	29	-47	-27	4.34
L medial cerebellum	1110	-17	-53	-38	4.38
R anterior insula	1022	30	14	12	4.81
L angular gyrus	550	-29	-71	34	3.93
*Pseudowords > phrases*					
R superior frontal gyrus	10,583	16	32	38	5.19
L precuneus	5091	-13	-51	39	4.92
L lateral occipital cortex	2357	-39	-83	2	5.07
R postcentral gyrus	592	25	-28	39	4.46
R angular gyrus	566	43	-66	27	4.33
L angular gyrus	458	-56	-58	23	4.09

#### Lateralization

The above analyses established the sensitivity of mROI defined by multivariate analysis of areas showing high inter- and intra-subject reliability. In this analysis we compared laterality indices between the multivariate and univariate ROI as a measure of face validity (Wilson et al. 2017). This is based on neuropsychological evidence that it is the left hemisphere that primarily houses neural tissue critical for language, damage to which leads to aphasia (Alexander 2003; Damasio 1992, 1998), as well as meta-analysis of functional neuroimaging studies showing that more activations for semantic processing are reported in the left than right hemisphere (Binder et al. 2009).

**Fig. 5:**
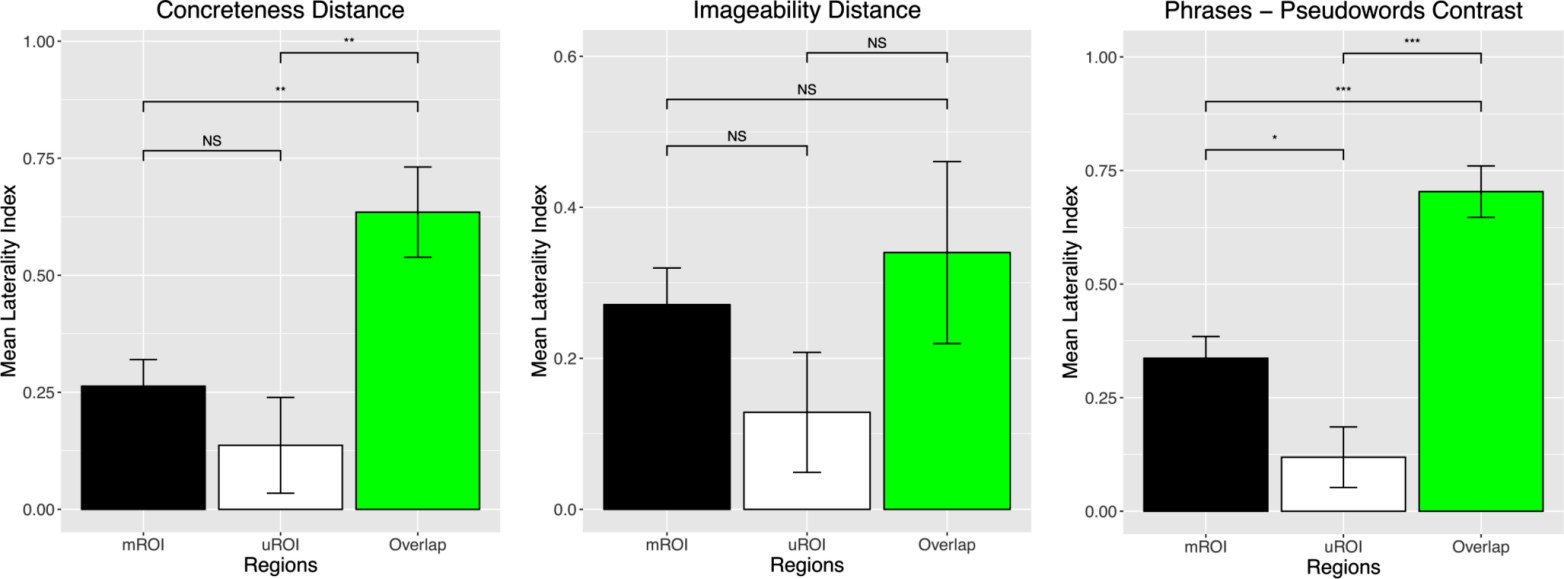
Laterality index comparisons among the two ROI and their overlap. Direct comparisons between the ROI only showed a significant difference for the phrases – pseudowords contrast, with the mROI showing greater left-lateralization then the uROI. The overlap of the ROI showed significantly greater left-lateralization for all conditions except imageability distance. NS = not significant, *p < 0.05, ** *p* < 0.005, *** *p* < 0.001

For the two studies using single-word stimuli, the mROI results showed numerically greater left-lateralization than the uROI results, but this difference was not statistically significant (Fig. 5). Results from Study 3 using multi-word stimuli, however, did show significantly greater left-lateralization within the mROI than the uROI. For two of the three studies (Studies 1 and 3), the area of overlap between the ROIs showed significantly greater left-lateralization than either of the other ROI alone. Also, as pointed out by an anonymous reviewer, visual inspection of Fig. 5 suggests that if a LI of 0.2 were used as a cutoff, the uROI would be shown to yield bilateral effects in all three cases.

## Discussion

The overall motivation for this study was to determine the feasibility of using a multivariate localizer, defined in terms of brain areas showing high reliability across stimuli and participants, to reveal brain areas representing aspects of language. This contrasts with a widely used approach of defining language areas in terms of a univariate localizer contrast of sentences > pseudowords. The multivariate approach is easily amenable to defining stimuli in terms of different aspects of language. The current study focused on semantics, but phonological measures or even syntactic measures with sentence stimuli could also be used. We expected that the multivariate approach would be more sensitive and inclusive than the univariate approach, including areas beyond peri-sylvian cortex, while also maintaining face validity for language, as measured by overall left-lateralization. We found that neural activation patterns associated with semantic dissimilarity patterns in the stimuli (defined in terms of pair-wise semantic distances between stimulus words) were significantly stronger for areas falling within the mROI than areas in the uROI. This was true across two datasets and two different metrics of semantic distance. The same pattern also held for results from a third dataset where the activations within the different localizers were defined in terms of a univariate contrast between meaningful article-noun-noun phrases and pseudowords. In each dataset, the top 20% of voxel parameter estimates from each ROI were greater in the multivariate than the univariate case. The pattern of lateralization effects generally tracked with the magnitude effects just described, except that the greater left-lateralization for the mROI than uROI was only significant for the univariate contrast. Interestingly, in all cases the spatial overlap between the mROI and uROI significantly showed the greatest magnitude of response and greatest left-lateralization.

### Localizers and regions of interest

For purposes of this study, we have used the terms “localizer” and “regions of interest” interchangeably. Strictly speaking, however, they are not the same. A localizer is a condition in the experiment used to create or define regions of interest. Here our localizer was defined based on representational fidelity analysis of data from Study 1, where input data consisted of neural responses to word events relative to an implicit fixation baseline. As implemented previously (Rothlein et al. 2018), the fidelity analysis reveals areas where representational geometries of neural responses to stimuli are consistent across runs and participants. This formed our mROI. Critically, the fidelity analysis is based exclusively on neural RDM, whereas the subsequent standard RSA analyses (Studies 1 and 2) compared neural RDM to predicted RDM. The predicted RDM were based on pair-wise stimulus differences in either concreteness (Study 1) or imageability (Study 2). Study 3 was distinct in that it was based on a univariate contrast between meaningful phrases and length-matched sets of pseudowords.

The comparison localizer was based on a contrast of sentences > pseudowords, as documented in Fedorenko et al. (2010). This formed our uROI. We projected that volume to the nearest cortical surface and displayed the outline in Figs. 3 and 4. In general, the uROI encompasses peri-sylvian cortex, while the mROI (Fig. 2 and outlined in black in Figs. 3 and 4) includes only some peri-sylvian cortex with much additional extra-sylvian cortex. The previous studies defining the uROI reflect a strong commitment to the idea that brain areas falling within the uROI should be interpreted as language-responsive cortex, while areas falling outside the uROI are responsive to functions other than language (Fedorenko and Shain 2021). Indeed, we cannot rule out the possibility of additional functions beyond language in the areas highlighted by the overlap of the mROI and RSA results in the current study. However, we feel that demonstrating the presence of language representations 2 in areas showing high levels of reproducibility beyond those of the uROI is an important step in achieving a more complete understanding of the neural distribution of language.

The observation that the overlap between the mROI and uROI showed the greatest magnitude of responses and left-lateralization raises the possibility that the optimal localizer would combine multivariate and univariate definitions. In considering this possibility, it is worth emphasizing the two distinct factors that went into defining the ROIs: reliability and domain-relevance. The mROI was focused on reliable voxels (for a different approach to reliability-based voxel selection, see Tarhan and Konkle 2020) at the level of multi-voxel patterns. The uROI was also derived from activations found to be consistent across numerous subjects, but based on univariate rather than pattern analysis. The domain-relevance comes both from the task being performed and from the factors being analyzed. For Study 1, the task was familiarity judgment and the stimulus factor being analyzed was concreteness. The mROI was based on data from participants performing the word familiarity judgment task, while the uROI came from separate datasets analyzed using a contrast of reading sentences > pseudowords. Because pseudowords also contain valid phonology due to being pronounceable, focusing analysis on the uROI risks excluding areas responsive to phonology. Therefore, while focusing on the area of overlap between the ROIs would likely yield higher magnitude effects and greater left-lateralization than from either ROI alone, this would come with a risk of excluding voxels relevant to one localizer more than the other.

To better understand the nature of the overlap between the ROIs, we note that the overlap of the mROI and uROI represents a greater percentage of the uROI volume (13.5%) than the mROI volume (8.1%). This also holds true when separately considering the left hemisphere (uROI: 19.0%, mROI: 9.5%), and to a lesser extent for the right hemisphere (uROI: 8.3%, mROI: 6.3%). The mROI was defined using the same data as was analyzed using RSA in Experiment 1. Therefore, while the conditions being analyzed were distinct, it is possible that the definition of the mROI could have been over-fit to the data, potentially inflating the overlap between the mROI and the RSA results. The most rigorous test we knew of for addressing this possibility was to apply the ROIs to new data derived from different tasks and analyses, which is what we did in Experiments 2 and 3. Also, the strong claim being made about the uROI is that it is relevant to language in general (Fedorenko and Shain 2021). Therefore, if it is indeed less relevant to the word recognition task (Exp 2) or meaningfulness judgment task (Exp 3), that would call into question the interpretation of the uROI that is preferred by the originators of that localizer.

### Semantics RSA

The semantic RSA results for Studies 1 and 2 are shown in Fig. 3A and B. The results were obtained from a partial correlation analysis. Concreteness was the semantic factor of interest in Study 1, where each word has a rated concreteness value (Brysbaert et al. 2014). The stimulus-stimulus distance matrix was defined as the absolute value of the difference in concreteness between each pair of words in the stimulus set. Stimulus-stimulus distance matrices defined in terms of phonological and orthographic edit distance measures were used to partial out effects of phonology and orthography.

The whole-cortex searchlight RSA results for stimulus geometries defined in terms of concreteness distance reflect areas previously found to be associated with semantics in functional neuroimaging studies using univariate analysis (Binder et al. 2009), such as the IFG pars orbitalis, posterior cingulate, angular gyrus, and middle temporal gyrus (including anterior regions), all more extensive on the left. Additionally, areas previously associated with the task-positive or multiple-demand network (Duncan 2010; Fox et al. 2005), such as the middle and superior frontal gyri and pre-SMA, were also significantly associated with concreteness. While it may seem surprising that the same analysis focused on semantics would reveal areas associated not only with semantics but also those associated with more domain-general task difficulty effects, other studies have shown that neural areas responding to task difficulty (Graves et al. 2017) can also contain information sufficient to decode word stimuli as being of either high or low imageability (Mattheiss et al. 2018).

Additional neural associations with concreteness were found in the superior temporal gyrus extending to the supramarginal gyrus. Activation in these areas has previously been associated with processing phonology (Graves et al. 2008; Vigneau et al. 2006), and damage to the posterior superior temporal and supramarginal gyri has been shown to impair the ability to access phonological word forms (Buchsbaum et al. 2011; Pillay et al. 2014). Phonological and orthographic distance measures were included in the partial correlation analysis of concreteness. It is possible, however, that some neural areas may compute representations that are a blend of, for example, phonological and semantic information. Such blended representations could be useful as intermediaries for mapping between word sounds and their corresponding meanings. These areas would presumably remain despite factoring out correlations with phonology due to their correlation with semantics. Indeed, the calculation of such blend states has been shown in artificial neural networks to be reflected in hidden unit representations that accomplish mappings between specified inputs and outputs (Harm and Seidenberg 1999; Plaut et al. 1996; Seidenberg and McClelland 1989).

The different ROIs captured distinct parts of the RSA results for concreteness. The mROI overlapped with the RSA results in the inferior and middle frontal gyri, medial areas in posterior cingulate and pre-SMA, and left AG. Distinct from this were areas captured by the uROI in bilateral IFG, along with superior and middle temporal gyri. In general, the mROI overlapped areas within peri-sylvian cortex but also beyond it to include extensive extra-sylvian areas. Many of the uROI areas are known to be critical for language based, for example, on studies of aphasia (Alexander 2003; Damasio 1992, 1998). However, many of the mROI areas, such as the angular and supramarginal gyri, are also known to be critical for language (Buchsbaum et al. 2011; Pillay et al. 2014; Seghier 2013, 2023). This suggests that using both ROIs could provide a more complete and robust picture of how aspects of language are neurally distributed.

Further evidence for the potential usefulness of a combined approach using both ROI comes from the observation that the areas of spatial overlap between the ROI showed significantly greater parameter estimates than either of the individual ROI alone. This was true for all three studies. The overlap area also showed greater left-lateralization in two of the three studies. Note that, while the mROI consisted of a larger number of voxels than the uROI, their overlap had the smallest volume, ruling out the possibility that advantages for the mROI were due only to having a larger volume. The exact source of the sensitivity of the overlap area to activation and lateralization across the language tasks is less clear from this study alone. We note, however, that it selectively encompasses areas more recently thought to be the core neural regions where damage leads to aphasia. This includes the posterior part of the opercular IFG, where damage extending to underlying white matter leads to Broca’s aphasia (Dronkers et al. 2007), especially when it includes the anterior termination of the arcuate fasciculus (Gajardo-Vidal et al. 2021). Similarly, overlap areas in posterior middle and superior temporal gyri are areas where damage leads to Wernicke’s aphasia (Binder 2015). Damage to areas of mid and anterior middle temporal gyrus have also been linked to deficits in word retrieval with more anterior damage (Schwartz et al. 2009) and to resistance to language recovery in aphasia when damage includes the middle parts of middle temporal gyrus (Wilson et al. 2023).

The RSA results for imageability were largely similar to those from concreteness. This was expected based on the similarity of the two factors. There were, however, also some differences. While the distinct nature of the datasets did not lend itself to a direct statistical comparison, qualitatively we note the extension of the imageability RSA results into additional parts of the superior frontal gyrus, bilateral inferior parietal lobule extending into superior parietal lobule, and left-lateralized ventral temporal cortex. The ventral temporal cortex result is particularly intriguing considering that all three tasks involved reading. The concreteness RSA produced a much smaller ventral temporal cortex result that did not overlap with the mROI, while the univariate phrases – pseudowords contrast also resulted in ventral temporal cortex activation that appeared similar in extent and location to the imageability RSA result. This area within the fusiform gyrus is spatially intermediate between the parahippocampal gyrus, an area reliably associated with semantic processing (Binder et al. 2009), and the more posterior visual word form area (Dehaene and Cohen 2011; Dehaene et al. 2005). A posterior-to-anterior gradient for words in the ventral temporal lobe would be consistent with previous findings (Vinckier et al. 2007). The fact that the ventral temporal semantic result found here lies proximal to the parahippocampal gyrus, however, suggests that it may reflect an intermediary mapping between word form and meaning (Devlin et al. 2006; Liuzzi et al. 2015), rather than a gradient toward increasingly familiar stored word forms, as suggested previously (Vinckier et al. 2007).

#### Univariate contrast for semantics

Results from the univariate contrast of meaningful phrases compared to length-matched pseudowords should be interpreted with caution. We note that it was not a main condition of interest in the original study and was only used here because it was somewhat analogous to the sentences > pseudowords contrast that defined the uROI. Also, the pseudoword condition appeared to be easier than the phrase condition. The task involved making meaningfulness decisions to phrases with real words, while the pseudoword condition required participants to simply press a button to indicate if the phrase consisted of pseudowords. Thus, the phrase condition required at least two cognitive processes: (1) recognizing that the phrase was made of real words, and then (2) determining phrase-level meaning. The pseudoword condition only required recognition that it was made of pseudowords. The presence of additional processing demands in the phrase condition is entirely consistent with the behavioral results, in which performance on the pseudoword condition was significantly more accurate and on average 95.1 ms faster than the phrases condition. This renders interpretation of the results a bit more complex than for single-word recognition tasks in which task demands were more closely matched for words and pseudowords. In such cases, words are typically responded to more quickly and accurately than pseudowords (Balota et al. 2004; Evans et al. 2012). Despite this ambiguity in determining whether the activations reflect differences in processing content or task difficulty, it is striking that activations within the ROI continue to show the same pattern of overlap > mROI > uROI, along with the same order of significant differences in left-lateralization. This is consistent with the finding that areas responding to task difficulty can also show patterns of activation related to specific types of content such as semantics (Mattheiss et al. 2018; Zhang et al. 2023).

### Lateralization and face validity

The lateralization index (LI) has been used previously as a way to quantify face validity of neural results from language tasks (Wilson et al. 2017). As noted in the Results, visual inspection of Fig. 5 shows that if a threshold of 0.2 were applied to the LI, that would separate the mROI from the uROI results in all three experiments. The results within the uROI were below 0.2, and therefore bilateral across the board, while results within the mROI were above 0.2, and therefore left-lateralized overall. By comparison, the two language mapping paradigms of the four tested by Wilson et al. (2017) that were judged to have the highest validity also had a LI greater than 0.2, while the others had a LI of 0.2 or below. This suggests that the uROI yielded bilateral language effects for all three studies, and therefore has less face validity than the mROI.

### Clinical implications

As alluded to in the Introduction, there are also clinical implications for how language-responsive cortex is defined. Several studies using the sentences > pseudowords contrast have sought to draw a bright line between what cortex can and cannot be considered to be specifically processing language (Fedorenko et al. 2012; Grand et al. 2022; MacGregor et al. 2022; Pereira et al. 2018; Schrimpf et al. 2021). Yet the organization of brain function also incorporates cases in which the same patch of neural tissue can carry out multiple functions (Poldrack 2010; Price and Friston 2005). Indeed, univariate analysis has shown that entire networks can change their response to which condition is being activated simply based on which condition is more difficult (i.e., longer response times and lower accuracies). Specifically, pseudowords have been shown to activate default mode areas typically associated with semantics when they were the easier condition compared to words (Graves et al. 2017). Yet multivariate analysis showed that even when activating for pseudowords, those same networks contained semantic information sufficient for decoding whether a high or low imageability word was being read (Mattheiss et al. 2018). Therefore, a strong commitment to strict modularity that excludes multi-functional cortex from consideration as language cortex risks missing areas that, for example, integrate linguistic and visual information.

This concern is more than hypothetical, particularly within the ventral temporal lobe. For example, an analysis of areas of brain tissue resection associated with development of anomia following surgery for treatment of focal epilepsy significantly implicated the left ventral temporal lobe, centered on the mid-fusiform gyrus and extending laterally to the inferior temporal gyrus (Binder et al. 2020). Notably, that area is included in our mROI but not in the uROI. Therefore, in the quest to identify language-specific cortex, the sentences > pseudowords contrast risks missing areas that are critical to basic language function such as naming.

The fact that the most robust results were derived from the overlap between the mROI and uROI raises the possibility that those areas are most important to preserve in a resection, followed by areas within the mROI. Clearly the time involved in acquiring scans is also a limitation when gathering data for clinical use. More work will be needed to determine the minimum scan time necessary to establish representational fidelity maps for the mROI used here (for recent progress in this direction with univariate analysis, see Lee et al. 2024). We suggest that a combined approach is promising for balancing inclusivity from the mROI with specificity from its overlap with the uROI.

### Limitations and future directions

The current study is a first step toward an ultimate use case of defining brain areas in a manner that reveals those most reliably associated with language, and that can reveal different aspects of language (semantics, phonology, syntax, and orthography in cases of reading) rather than treating language as a monolithic construct. Clearly additional steps are needed to achieve that larger goal. For example, the current study only focused on semantics. While the univariate localizer that the uROI was based on includes semantics, it presumably includes syntax as well, since the length-matched pseudoword condition is aimed at subtracting out phonology and orthography. Phonology is, of course, also a critical aspect of language, and a more complete demonstration of the utility of the multivariate approach to language localization will need to demonstrate sensitivity to phonology as well. In principle the same is true of syntax for connected speech or text. In practice, however, the most common language complaint following, for example, surgical excision for epilepsy, is word retrieval difficulties or anomia (Hamberger 2015). Evidence pointing to a combination of semantic and phonological impairments as primary sources of anomia (Dell et al. 1997; Goldrick and Rapp 2002) suggest that it may be most fruitful to focus on highlighting neural areas most reliably associated with phonology, and its integration or overlap with semantics, as important next steps.

We should also acknowledge that neural areas involved in processing phonology, as well as other language functions, are not only cortical but involve subcortical structures as well. For example, the thalamus has been shown to be involved in several aspects of language, including production and comprehension (Crosson 2013; Janacsek et al. 2022; Llano 2013). Likewise, the cerebellum is involved in numerous aspects of language, including reading (Fiez 2016). Our choice to focus on cortex follows best-practice guidelines laid out by Kriegeskorte et al. (2006) in their development of searchlight analysis. Also, our fMRI acquisition field of view did not always include full coverage of the cerebellum. Considering the evidence for involvement of subcortical structures in language, however, including subcortical structures remains fertile ground for yielding potentially insightful future studies.

Here we have focused on what we feel is the most salient difference between the two ROIs under consideration. The multivariate approach allows for a focus on representational content, while the univariate approach is a contrast between two conditions, both of which are arguably language-relevant since the control condition also includes pronounceable phoneme sequences. However, other differences may also be contributing to the divergent results between the ROIs. For example, the fMRI data on which the ROIs were based are different, as they were derived from the use of different tasks, different statistical thresholds were applied, and there were likely different signal-to-noise ratios arising from the use of different voxels sizes between the studies (27 mm^3^ in the current study, compared to 38.4 mm^3^ in Fedorenko et al. 2010). More definitive conclusions could be drawn from directly comparing the two approaches in a single study in which more experimental parameters were held constant.

It is also an inherent limitation of functional recording techniques such as fMRI that they cannot reveal which brain areas are critical or necessary for the function in question. Establishing such a relationship requires additional techniques such as brain lesion-behavior mapping (Vaidya et al. 2019). Several important lesion-deficit studies have been performed that focus on relevant aspects of language such as phonological access (Pillay et al. 2014), semantic retrieval and comprehension (Dronkers et al. 2004; Schwartz et al. 2009), or both (Dickens et al. 2019). To our knowledge, however, no direct comparison of multivariate language localizers with multivariate lesion-behavior mapping has been performed. Such a comparison would be a critical next step in determining which functionally mapped neural areas, inclusive for semantics, phonology, etc., are also necessary for those functions. We predict that areas revealed by such an analysis, when focused on semantics as in the current study, will reveal areas highlighted here in the overlap between the mROI and uROI as critical for linguistic function, lending additional weight to the conclusion that those areas are particularly critical for representing aspects of semantics in language.

## Conclusions

Overall, results of the current study suggest that the multivariate ROI, defined in terms of areas showing reliability across runs and participants, was particularly promising for revealing functional patterns related to semantics. This was shown by tests of sensitivity and face validity. Sensitivity was shown by finding stronger neural associations within the mROI compared to the uROI. Face validity was shown by generally greater left-lateralization within the mROI compared to the uROI. Across the board, however, the greatest levels of sensitivity and validity were found in areas where the mROI and uROI overlapped. This suggests that using both multi- and univariate localizers would be an especially promising avenue to explore for neurally localizing the multiple components of language.

### Electronic supplementary material

Below is the link to the electronic supplementary material.

Supplementary Material 1

Supplementary Material 2

## Footnotes

1 To gain a sense of the spatial distribution of the top 20% voxels, we also restricted the representational fidelity results to the top 20% voxel intensities. No analogous calculation could be done with the uROI because only label rather than real-value results were available. A total of 52 voxels overlapped between the top-20% mROI and the uROI.

2 The scale of the y-axis is larger in Fig. 4 compared to Fig. 3. This is because the values in Fig. 3 are based on Spearman (rho) correlations, which are bounded between 0 and 1, whereas Fig. 4 is based on beta weights from ordinary least squares regression that are not restricted to the same 0 to 1 range.
