# Supplementary material for: An inclusive multivariate approach to neural localization of language components
Source: Brain Struct Funct. 2024 May 2;229(5):1243–63. doi: 10.1007/s00429-024-02800-9 (PMC11147878; doi:10.1007/s00429-024-02800-9)
Supplement: Supplementary file 1 — Supplementary Material 1 [file 429_2024_2800_MOESM1_ESM.pdf]

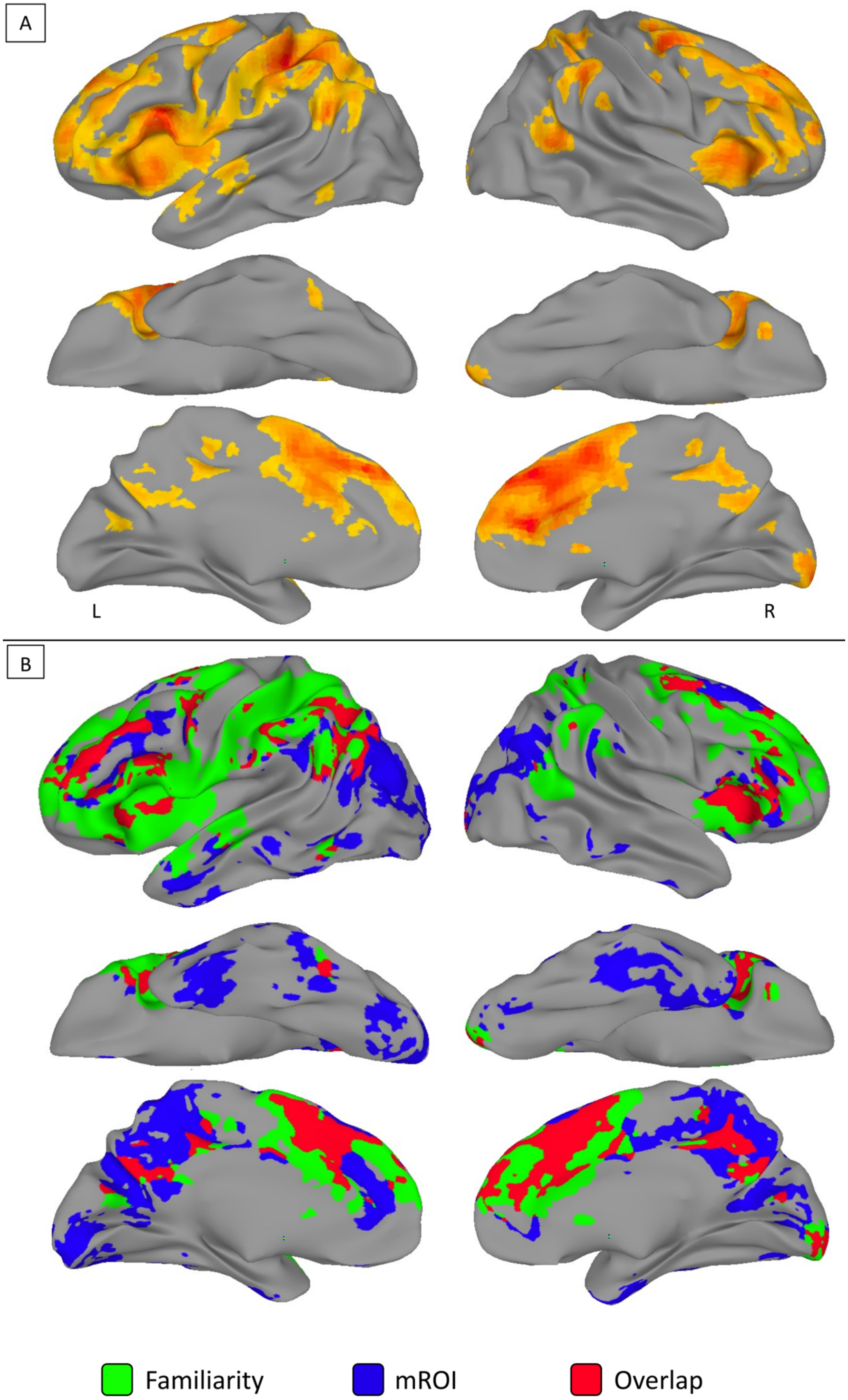
A
L
R
B
Familiarity
mROI
Overlap

Supplementary Figure 1: Panel A shows RSA searchlight results from Study 1 for areas significantly associated with the pattern of word familiarity judgments performed by each participant. Panel B shows the spatial distribution of thresholded results from the familiarity judgment analysis (panel A, green in panel B), the representational fidelity analysis that formed the multivariate region of interest (mROI, blue), and their overlap (red).
