# Supplementary material for: An inclusive multivariate approach to neural localization of language components
Source: Brain Struct Funct. 2024 May 2;229(5):1243–63. doi: 10.1007/s00429-024-02800-9 (PMC11147878; doi:10.1007/s00429-024-02800-9)
Supplement: Supplementary file 2 — Supplementary Material 2 [file 429_2024_2800_MOESM2_ESM.pdf]

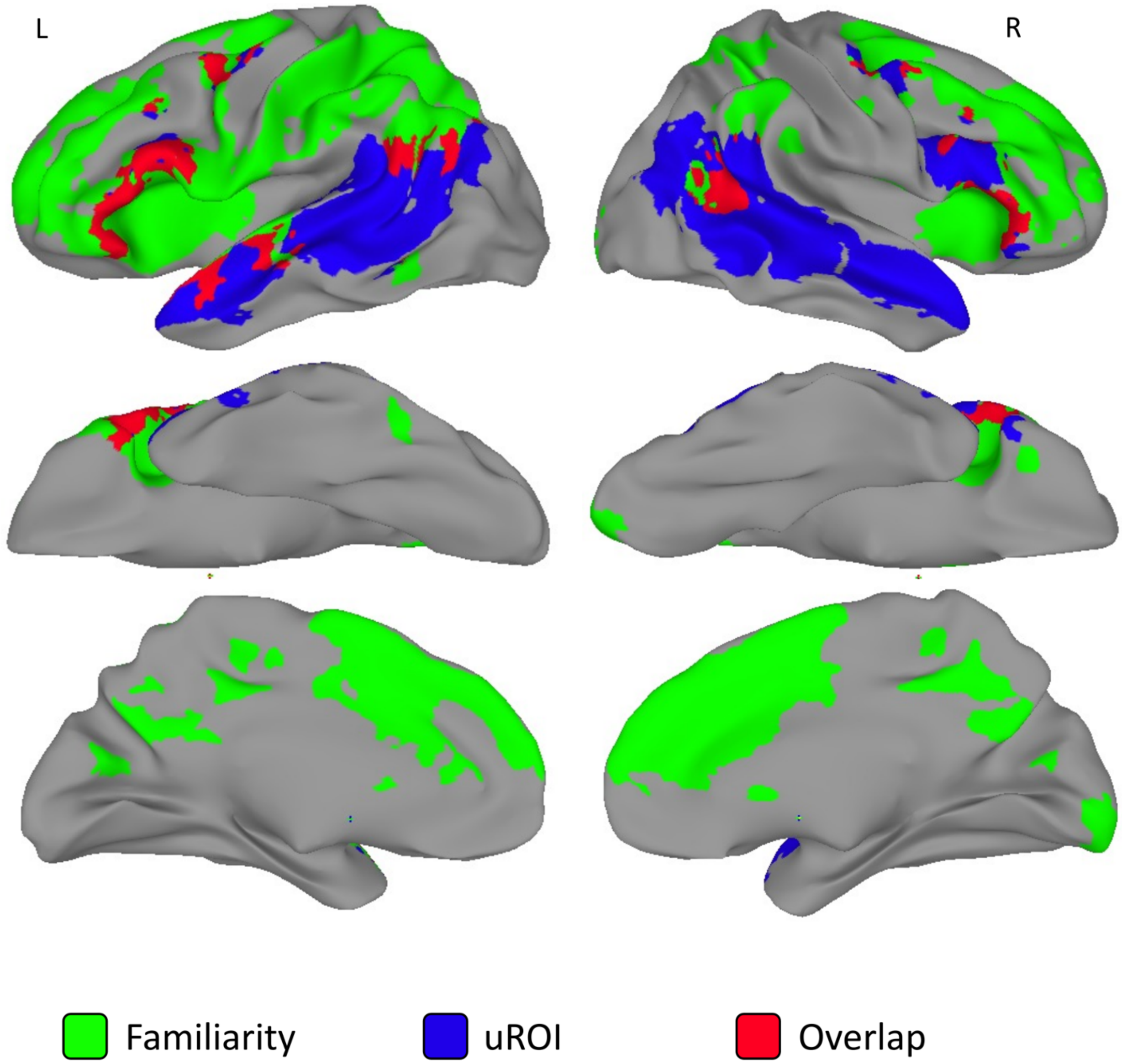

Supplementary Figure 2: Color coded spatial distribution of thresholded results from the familiarity judgment analysis (green), the sentences > pseudowords analysis from Fedorenko et al. (2010) that formed the univariate region of interest (uROI, blue), and their overlap (red).
